# Diabetic Cardiomyopathy: Role of Cell Death, Exosomes, Fibrosis and Epicardial Adipose Tissue

**DOI:** 10.3390/ijms25179481

**Published:** 2024-08-31

**Authors:** Antonella Galeone, Alessia Annicchiarico, Cinzia Buccoliero, Barbara Barile, Giovanni Battista Luciani, Francesco Onorati, Grazia Paola Nicchia, Giacomina Brunetti

**Affiliations:** 1 Department of Surgery, Dentistry, Pediatrics and Gynecology, Division of Cardiac Surgery, University of Verona, 37129 Verona, Italy; antonella.galeone@univr.it (A.G.); giovanni.luciani@univr.it (G.B.L.); francesco.onorati@univr.it (F.O.); 2 Department of Biosciences, Biotechnologies and Environment, University of Bari Aldo Moro, 70125 Bari, Italy; alessia.annicchiarico@uniba.it (A.A.); cinzia.buccoliero@uniba.it (C.B.); barbara.barile@uniba.it (B.B.); graziapaola.nicchia@uniba.it (G.P.N.)

**Keywords:** diabetic cardiomyopathy, apoptosis, autophagy, pyroptosis, ferroptosis, fibrosis, exosomes

## Abstract

Diabetic cardiomyopathy (DCM) represents one of the typical complications associated with diabetes. It has been described as anomalies in heart function and structure, with consequent high morbidity and mortality. DCM development can be described by two stages; the first is characterized by left ventricular hypertrophy and diastolic dysfunction, and the second by heart failure (HF) with systolic dysfunction. The proposed mechanisms involve cardiac inflammation, advanced glycation end products (AGEs) and angiotensin II. Furthermore, different studies have focused their attention on cardiomyocyte death through the different mechanisms of programmed cell death, such as apoptosis, autophagy, necrosis, pyroptosis and ferroptosis. Exosome release, adipose epicardial tissue and aquaporins affect DCM development. This review will focus on the description of the mechanisms involved in DCM progression and development.

## 1. Introduction

Diabetic cardiomyopathy (DCM) represents one of the typical complications associated with diabetes. It has been described as anomalies in heart function and structure, with consequent high morbidity and mortality. However, the pathophysiological mechanisms of DCM can be different according to the type of diabetes. It is important to remember that the two main types of diabetes are type 1 diabetes (T1D) and type 2 diabetes (T2D). The first is characterized by insulin deficiency [1], whereas the latter is characterized by insulin resistance [2]. Consistently, DCM is associated with left ventricular mass increase [3] that in T1D may be due to the younger age of disease development, whereas in T2D, DCM is independent of factors such as race and obesity [4]. In the attempt to deepen the mechanisms, researchers focus on insulin signaling, which involves two interacting pathways: the MAPK pathway and the insulin receptor substrate 1 (IRS-1) pathway [5]. Obviously, in T2D, insulin resistance alters the balance, shifting it in favor of the MAPK pathway with consequent effects on cell metabolism and growth leading to cardiac fibrosis and diastolic dysfunction.

**Figure 1:**
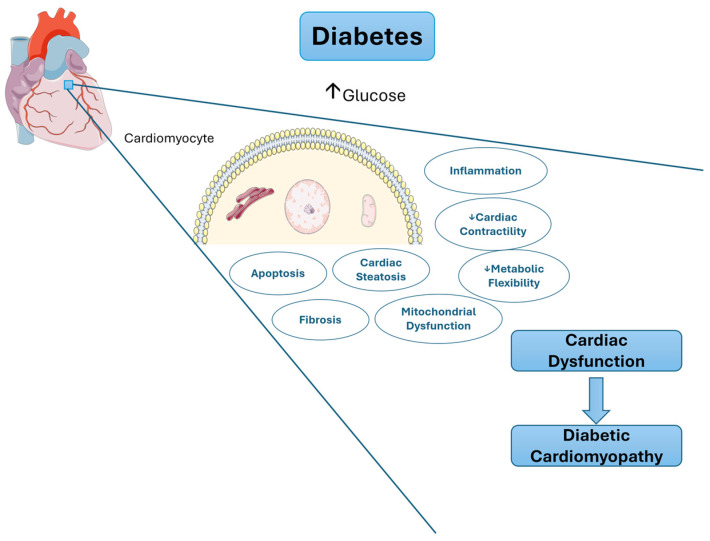
Diabetic cardiomyopathy mechanisms. Figure was generated using Servier Medical Art, provided by Servier, licensed under Creative Commons Attribution 3.0 Unported license.

DCM development can be described by two stages; the first is characterized by left ventricular hypertrophy and diastolic dysfunction, and the second by heart failure (HF) with systolic dysfunction [6]. The proposed mechanisms involve cardiac inflammation, oxidative stress, advanced glycation end products (AGEs) and angiotensin II (Ang-II) [7,8]. Furthermore, different studies have focused their attention on cardiomyocyte death through the different mechanisms of programmed cell death, such as apoptosis, autophagy, necrosis, pyroptosis and ferroptosis [9,10]. In this review, we will detail the mechanisms associated with these processes (Figure 1).

## 2. Cardiomyocyte Cell Death

### 2.1. Cardiomyocyte Apoptosis

Cardiomyocyte apoptosis may occur in the initial stage of DCM development and is linked to myocardial hypertrophy and HF. The mechanisms reported endoplasmic reticulum (ER) stress as a crucial event for cardiomyocyte apoptosis in DCM. Positive effects have been reported for chlorogenic acid and hydrogen sulfide to counteract ER stress using in vitro and in vivo models. Oxidative stress in myocardial ER can be activated by permanent hyperglycemia, resulting in caspase activation [11,12,13]. Consistently, high glucose levels have been linked to caspase-8 and -9 in cardiomyocytes in neonatal rats [14].

An additional factor affecting cardiomyocyte apoptosis in DCM is represented by chronic inflammation, which leads to the increased production of nitric oxide (NO) and sustains the expression of proto-oncogenes with the consequent development of ventricular diastolic dysfunction [15]. The altered levels of glucose in DCM patients’ blood determine the activation of leukocytes together with the recruitment of activated monocytes, neutrophils and macrophages. Consequently, the levels of pro-inflammatory cytokines are also increased. Consistently, high levels of Interleukin (IL)-1β, IL-6, Tumor Necrosis Factor (TNF)-α and Tumor Growth Factor (TGF)-β1 lead to cardiomyocyte apoptosis and thus DCM [16]. Inflammation can be also exacerbated by increased levels of ROS and NLR family pyrin domain containing 3 (NLRP3) inflammasome. Consistently, the dysregulation of the latter is associated with an immunomodulatory response in DCM; in contrast, NLRP3 silencing improves cardiac remodeling in diabetes and alters cardiac function [17].

A key role has also been attributed to intracellular Ca^2+^ levels, which are important for cardiomyocyte contraction. Interestingly, the abnormal gene transduction of Ca^2+^-ATPase has been demonstrated in diabetic rats, which displayed a reduced uptake of Ca^2+^ in the sarcoplasmic reticulum together with a reduction in the Na^+^-Ca^2+^ transporter in the cardiomyocyte membrane, leading to an increase in Ca^2+^ in the cells, with a consequent increased duration of the action potential and a shortened systolic phase, together with a prolonged diastolic phase [18,19,20]. Ca^2+^ overload is also associated with an increased uptake by mitochondria, which is linked to cardiomyocyte apoptosis [21].

### 2.2. Cardiomyocytes Pyroptosis

Pyroptosis and apoptosis represent two distinct types of programmed cell death with some similarities [22]. In detail, pyroptosis is associated with membrane destruction, cell swelling, intact nucleus, inflammation, pore formation, pyroptotic body formation and the activation of caspase-1, caspase-4, -5, -11 and -12, whereas apoptosis is characterized by apoptotic body formation and caspase-2, -7 and -10 activation [23,24,25]. Pyroptosis can sustain DCM development through the involvement of different pathways, and myocardial cells can die by pyroptosis to speed up DCM development. Different pathways can be involved in DCM pyroptosis, such as NF-κB/NLRP3, ROS and Nuclear factor erythroid 2-related factor 2 (Nrf-2) [26,27,28,29,30]. Hyperglycemia leads to elevated ROS production, promoting the binding of TXN1P to NLRP3, and consequently the activation of the inflammasome, which is also a crucial mediator of DCM progression [31].

### 2.3. Autophagy

The word autophagy arises from Greek and characterizes the self-degradation and recycling of cellular components [32]. In heart homeostasis, autophagy represents a dynamic process leading to eliminated misfolded proteins, damaged organelles and cellular debris [33,34]. It is a tightly regulated process fundamental for cellular adaptation, survival and renewal. Consistently, hyperglycemia impairs autophagy by inhibiting autophagosome formation and maturation, with the consequent accumulation of unuseful material in cardiomyocytes, thus impairing cardiac activity in diabetes [35,36]. Oxidative stress also impairs autophagy through the disruption of redox-sensitive pathways. An additional modulator of autophagy is represented by inflammation, which contributes to the alteration of cardiac remodeling dysfunction in DCM [37,38].

A key role in autophagy has been demonstrated by noncoding RNAs, including microRNAs (miRNAs), long noncoding RNAs (lncRNAs) and circular RNAs. LncRNAs have a length of about 200 nucleotides, lack protein-coding properties, and regulate gene expression and different cellular processes [32,39]. Among these, the lncRNA Diabetic Cardiomyopathy-Related Factor (DCRF) is involved in DCM pathogenesis [40]. This factor affects gene expression and cellular activities in the cardiomyocytes of diabetic patients. Consistently, using a rat model of DCM, it has been demonstrated that DCRF knockdown led to diminished cardiomyocyte autophagy, ameliorated cardiac function and reduced myocardial fibrosis. Increased DCRF levels were linked to hyperglycemia [32]. An additional important lncRNA is represented by H19. Using a rat model, the role of H19 has been evaluated in DCM. In diabetic rats, overexpression led to reduced cardiomyocyte autophagy and increased left ventricular performance [41,42].

GAS5 is involved in the regulation of cellular growth and apoptosis. In DCM, GAS5 attenuated myocardial damage by stimulating cardiomyocyte autophagy through the modulation of the miR221-3p/p27 axis [43,44]. NEAT1 is a lncRNA localized in the nucleus that participates in nuclear body formation. It is involved in numerous cellular processes, such as nuclear structure maintenance and gene expression modulation. In diabetic rats, Neat1 overexpression determined increased serum myocardial enzyme levels, reduced superoxide dismutase concentration and cardiomyocyte viability, and augmented infarct size. In diabetic rats, enhanced Neat1 levels affected cardiac ischemia/reperfusion damage through the induction of autophagy [45,46].

miRNAs have been implicated in different cellular processes, both in health and disease. Their dysregulation has been implicated in different diseases including DCM. The role of miR-200a-3p in DCM has been investigated using a model of DCM realized with db/db mice. These mice displayed reduced levels of miR-200a-3p in the heart. Diabetic mice displayed the overexpression of this miRNA, with consequent improved autophagy and decreased myocardial injury, cardiac dysfunction, apoptosis, fibrosis and inflammation. In this model, the increased levels of miR-200a-3p are linked to DCM in T2D by modulating the Mst1/Sirt3/AMPK axis acting on the expression of *FOXO3* [47].

Another important miRNA is represented by miR-207; its role in T2D has been studied in DCM animal models. They displayed autophagy malfunction, enhanced cell apoptosis and affected miR-207 levels. In cardiomyocytes, miR-207 inhibited autophagy and enhanced apoptosis. Additionally, in cardiomyocytes, miR-207 targeted LAMP2, an important autophagy-related protein, to inhibit autophagy and promote apoptosis, thus promoting DCM in T2D [48,49].

The role of miR-30d has been evaluated in DCM, showing that it inhibited autophagy in rats by modulating the KLF9/VEGFA pathway [50].

Other authors reported that miRNA expression can be affected by glycemic management but cannot be reversed to control the condition [51].

### 2.4. Ferroptosis

Ferroptosis is an iron-dependent nonapoptotic cell death associated with lipid-peroxidation accumulation, leading to the release of extremely cytotoxic products such as malondialdehyde and 4-hydroxynonenal, which damage cell membranes, proteins and DNA [52]. Different mediators have been demonstrated to be involved, such as CD36, Nrf2 and Zinc finger antisense (ZFAS1). In detail, CD36 is a multifunctional receptor involved in lipid metabolism and transport, energy metabolism reprogramming and inflammatory response. CD36 expression increases in DCM cardiomyocytes.

The transcription factor Nrf2 modulates the transcription of different factors, such as scavenger receptors, antioxidant genes, and autophagic and transporter proteins [53]. In vivo, it works as an antioxidant stress regulator. In DCM, Nrf2 is associated with ferroptosis in cardiomyocytes [54]. It has been demonstrated that DCM could activate Nrf2 by blocking autophagy, as well as Nrf2-mediated iron overload and lipid peroxidation, that in turn activated ferroptosis in cardiomyocytes [55].

ZFAS1 is a new lncRNA involved in numerous diseases. Its upregulation, together with miR-150-5p levels and ferroptosis, can be found in high glucose-treated cardiomyocytes and DCM animal models [56]. Interestingly, Cyclin D2 can modulate miR-150-5p. Cyclin 2 overexpression inhibited ferroptosis, whereas its downregulation stimulated ferroptosis [56].

## 3. Aquaporins and DCM

Aquaporins (AQPs) are a ubiquitous family of water channels that mediate the transport of water and other small molecules across cell membranes [57]. The major AQPs in the human cardiovascular system are AQP1, expressed in arteries, endothelia, myocytes and vascular smooth muscle cells [58]; AQP3, AQP4 and AQP7, localized in cardiomyocytes; and AQP9, found in endothelial cells. 

Despite the widely recognized role of AQPs in cardiovascular physiology and pathology [58,59,60], very little is known about their role in DCM. AQP1 has been identified as an emerging protein involved in the pathophysiology of myocardial edema, coronary atherosclerosis [58] and also DCM, where its expression was found to be detrimentally reduced in high glucose-evoked cardiomyocyte injury [61]. It has been proven that the inhibition of miR-1306–5p upregulates AQP1 and ameliorates the derived injury. Similarly, AQP1 and AQP4 are likely to play a beneficial role in DCM resulting from a pharmacological study evaluating the effects of the antidiabetic treatment Empaglifozin (EMPA) in type 2 diabetic rats [61]. In this study, the administration of EMPA was found to be associated with beneficial cardioprotective effects, such as decreased fibrosis, apoptosis and edema, likely due to the upregulation of the water channels and, therefore, fine water balance control [61]. To our knowledge, these are the only studies that provide evidence of the involvement of water channels in diabetic cardiomyopathy. Further research is needed to better understand their contribution to this promising field of study.

## 4. DCM and Exosomes

Exosomes, small extracellular vesicles of 30–100 nm in size, have been studied for several years in different areas of research from tumors to cardiovascular diseases, prospecting their use in clinical applications [62,63,64,65,66,67,68]. Exosomes can carry proteins, RNAs (mRNA, miRNA and noncoding RNA) and DNA sequences of great interest. Exosomes are studied for their ability to act in intercellular communication. For example, noncoding RNAs released from tumor-derived exosomes induce the polarization of M1 (pro-inflammatory) macrophages to M2 (anti-inflammatory) phenotypes, enhancing the suppression of immune cells [69]. Of great interest is the therapeutic potential of exosomes in different diseases including T2D, cutaneous wound healing, kidney, ocular and Alzheimer’s disease [70,71,72,73,74,75,76,77]; in cancer therapy resistance to chemotherapy agents [78]; in drug delivery [79,80,81]; and in autoimmune diseases including rheumatoid arthritis (RA), systemic lupus erythematosus (SLE), T1D, Sjogren’s syndrome (SS), multiple sclerosis (MS), inflammatory bowel disease (IBD) and systemic sclerosis (SSc) [82]. Furthermore, exosomes promote rotator cuff tendon–bone healing [83] and cerebral ischemia repair [84]; improve the repair of diabetic ischemia of the hind limb [85] and tendinopathy [86]; and promote skin wound healing [70,87]. Moreover, the exosomes’ role in heart disease, including DCM, and related clinical studies and therapy has been investigated [88,89,90,91,92].

Chaturvedi et al. investigated the benefit of cardiosomes, exosomes released from cardiomyocytes, during physical exercise on the cardiovascular complications of diabetes [93]. The study focuses on the ability of exercise to reduce the levels of matrix metalloprotease 9 (MMP9) in db/db mice as models of T2D and on the underlying molecular mechanism. Notably, Chaturvedi hypothesized the release of specific cardiomyosome microRNAs (mir455, mir29b, mir323-5p and mir466), which, binding to the 3′ region of MMP9, downregulated its expression and mitigated extracellular matrix remodelling [93]. Moreover, Hirai et al. investigated the role of cardiosphere-derived exosomes in myocardial repair in pediatric cardiomyopathy [94]. Of note, cardiosphere-derived exosomes, enriched with miR-146a-5p, inhibit myocyte apoptosis and fibrosis, enhancing angiogenesis and cardiac activity after infarction [94]. Notably, Gan et al. studied the role of circulating extracellular vesicles isolated from the serum of mice fed either normal or high-fat diets in exacerbating myocardial ischemia/reperfusion injury [95]. Compared to the control, the intramyocardial injection of serum vesicles from the animals fed a high-fat diet significantly increased myocardial ischemia/reperfusion (MI/R) injury in mice [95]. This result was confirmed by the poor recovery of cardiac function, larger infarct size and increased death by apoptosis [95]. In contrast, the injection of vesicles from the animals fed a normal diet had an opposite effect in reducing myocardial ischemia/reperfusion injury [95]. Moreover, the intramyocardial injection of diabetic adipocyte vesicles and high glucose/high lipid-challenged nondiabetic adipocytes exacerbated MI/R damage. Notably, miR-130b-3p levels were significantly increased in all the above vesicles, and the intramyocardial administration of miR-130b-3p significantly increased MI/R injury in the nondiabetic mice, whereas miR-130b-3p inhibitors significantly attenuated MI/R injury in the diabetic mice [95]. Of great interest is the possibility of using stem cell-derived exosomes in treating cardiovascular diseases and in promoting cardiac repair, enhancing angiogenesis and reducing apoptosis [96].

Yu et al. reported that exosomes released from mesenchymal stem cells expressing high levels of GATA4 had cardioprotective capabilities, preserving cardiac contraction and reducing infarct size. In this regard, miR-19a was higher in cardiomyocytes and myocardia treated with exosomes derived from mesenchymal stem cells overexpressing GATA-4 than in those treated with exosomes derived from control mesenchymal stem cells [97].

In addition, it has been shown that exosomes, depending on the content released, can induce positive or negative effects on the myocardium [98,99]. In diabetic cardiomyopathy, high levels of miR-320 have been detected in cardiomyocyte-derived exosomes, adversely affecting the heart [100]. The use of an exosome secretion inhibitor, such as GW4869, could be a potential therapeutic strategy to mitigate exosome-mediated cardiac dysfunction in diabetic hearts [100,101,102]. Wang and colleagues demonstrated that exosomes released from diabetic cardiomyocytes contained detrimental substances, such as lower levels of Hsp20 than normal ones, implicated as a primary factor contributing to T1D- and T2D-induced organ damage including ventricular dysfunction, cardiac fibrosis and cardiomyocyte apoptotic death [101]. In addition to impaired cardiac function, exosomes released from diabetic cardiomyocytes can also mediate anti-angiogenesis events through the exosomal transfer of miR-320 into endothelial cells and embryonic development, since maternal exosomes in diabetes could cross the maternal–fetal barrier, promoting cardiac developmental deficits [102,103]. In this context, several studies have shown that cardiomyocyte-derived exosomes contain different mRNAs and miRNAs, proteins, and lipids, which can be released to adjacent cardiac endothelial cells, positively or negatively modulating their activity [102,104,105,106].

Exosomes derived from nondiabetic Wistar rat cardiomyocytes promoted the proliferation and migration of cardiac endothelial cells. In contrast, exosomes isolated from diabetic Goto–Kakizaki (GK) rat cardiomyocytes reduced the proliferation and migration of cardiac endothelial cells [107]. The contents of the exosomes isolated from diabetic GK cardiomyocytes and the exosomes from Wistar rats were investigated. Exosomes from diabetic GK cardiomyocytes had higher levels of miRNA-320 and lower levels of miRNA-126 and heat shock protein 20 (Hsp20) than exosomes isolated from nondiabetic Wistar rat cardiomyocytes [102]. miRNA-320 can be released to cardiac endothelial cells, downregulating the expression of IGF-1, Hsp20 and Ets-2 and negatively affecting the angiogenic role of the adjacent cardiac endothelial cells. In addition, Garcia and colleagues have shown that under conditions of glucose deprivation, immortalized H9C2 cardiomyocytes produce more exosomes whose cargo affects cardiac endothelial cell activity, inducing modification in the transcription of pro-angiogenic genes [108].

## 5. Cardiac Fibrosis

DCM is characterized by myocardial hypertrophy and fibrosis in the absence of coronary artery disease, hypertension, or valvular heart disease [109]. Cardiac fibrosis is a prominent feature of diabetic cardiomyopathy that increases myocardial stiffness and is associated with reduced diastolic function and systolic dysfunction that eventually leads to HF, and may promote arrhythmogenesis and a higher risk of sudden death [110]. Cardiac fibrosis is the consequence of the accumulation of the extracellular matrix (ECM) produced by cardiac fibroblasts [111]. Distinct types of myocardial fibrosis have been described, such as replacement fibrosis, interstitial fibrosis and perivascular fibrosis [111]. Replacement fibrosis is usually seen in myocardial infarction and refers to the formation of collagen-rich scar tissue in areas of myocardial necrosis [112]. Replacement fibrosis is the result of a reparative process following cardiomyocyte injury. Despite the absence of ischemic injury leading to myocardial necrosis in diabetic cardiomyopathy, abnormal metabolism represents a chronic myocardial cell injury and may induce cardiomyocyte apoptosis, leading to replacement fibrosis. Interstitial fibrosis refers to the deposition of the ECM in the endomysium and perimysium, while perivascular fibrosis indicates the expansion of periadventitial collagen in the cardiac microvasculature. In contrast to replacement fibrosis, interstitial and perivascular fibrosis are not related to cardiomyocyte death, but are rather the results of metabolic alterations, inflammation and oxidative stress. Cardiac fibroblasts are the main ECM-producing cells [113] and multiple pathways with a complex interplay are involved in the activation and proliferation of cardiac fibroblasts and the production of ECM proteins in diabetic cardiomyopathy, such as hyperglycemia, insulin resistance, AGEs, TGF-β, renin–angiotensin–aldosterone system (RAAS), and the imbalance between matrix metalloproteinases (MMPs) and tissue inhibitors of metalloproteinases (TIMPs) [109].

Hyperglycemia and insulin resistance promote the proliferation of cardiac fibroblasts and increase the production of ECM proteins. Experimental studies in vitro showed that a high-glucose environment stimulates cultured cardiac fibroblasts to synthesize large amounts of ECM proteins such as collagen and fibronectin [114]. Hyperglycemia also promotes the production of AGEs that bind to specific receptors on the cell membrane, induce the release of large amounts of reactive oxygen species (ROS) and activate nuclear factor-κ-gene binding (NF-кB), which is a transcription factor of various inflammatory factors such as TNF-α and IL-6 and regulates the expression of pro-fibrotic and hypertrophy-related genes [115]. AGEs also cause the cross-linking of myocardial collagen molecules to each other, leading to the loss of collagen elasticity, and subsequently to the reduction in myocardial compliance [116].

Insulin resistance significantly limits the utilization of glucose by cardiac cells, with a consequent shift towards the use of fatty acids for energy production [117]. The increased mitochondrial fatty acid uptake and β-oxidation may induce mitochondrial dysfunction and the intracellular accumulation of toxic lipids and lipid metabolites that may cause myocardial necrosis and fibrosis [118].

Hyperglycemia also induces the RAAS activation that contributes to the myocardial hypertrophy and fibrosis observed in patients affected by diabetic cardiomyopathy [119]. In patients affected by diabetes, there is an increase in the tissue expression and activity of Ang-II, which binds to angiotensin receptor-1 present on cardiomyocytes and cardiac fibroblasts and induces cell proliferation and collagen synthesis, causing cardiac hypertrophy and fibrosis. In vitro and in vivo studies showed that TNF receptor 1 signaling is necessary for the Ang-II-induced transcriptional upregulation of several fibrosis- and inflammation-related genes and the development of cardiac fibrosis [120]. The harmful effects of Ang-II are counterbalanced by the heptapeptide angiotensin-(1-7) that shows a protective role in the cardiovascular system by binding to the Mas receptor and inhibiting cardiac cells’ growth [121].

The balance between MMPs and TIMPs is essential for the regulation of ECM degradation. High glucose stimulation may lead to an imbalance in the synthesis and degradation of the ECM and collagen, thus promoting cardiac fibrosis. Experimental studies show that MMP-2 expression is downregulated in streptozotocin-induced diabetic mice, and it is associated with a reduction in collagen degradation and an increase in TGF-β and myocardial fibrosis [122]. Additionally, the blocking of the AT-1 receptor is associated with the normalization of MMP activity and the reduction in TGF-β levels and cardiac fibrosis [122].

The TGF-β signaling pathway regulates cell proliferation, differentiation and migration and gene expression, and is implicated in reparative and fibrotic processes. TGF-β transduces signaling through a type II receptor (TGFβR2) which is constitutively active on the cell surface. The binding of TGF-βs to the TGFβR2 recruits and phosphorylates type I receptor kinases (TGFβR1), which in turn phosphorylates intracellular transcriptional regulators, namely the receptor-activated Smads (R-Smads) such as Smad2 and Smad3. Activated R-Smads form complexes with the common Smad, Smad4, and translocate to the nucleus, where they regulate the transcription of target genes [123]. Smad6 and Smad7 are TGF-β antagonists, or inhibitory SMADs, that combine with active TGFβR1 and prevent Smad2/3 binding and activation. Besides the activation of the canonical Smad-dependent cascade, TGF-βs can also activate the MAPK family, including extracellular signal-regulated kinase 1/2 (ERK1/2), p38 MAPK and c-Jun amino-terminal kinase (JNK) signaling [124]. Many cell types, including cardiac fibroblasts, produce and secrete TGF-β when they are exposed to high levels of glucose [125]. TGF-β signaling is also triggered by increased levels of Ang-II, cytokines and ROS, which are able to activate local stores of TGF-β, promote the transcription and secretion of TGF-β isoforms, and induce the synthesis and externalization of TGF-β receptors on the cell surface. TGF-β signaling induces cardiac fibroblast proliferation and differentiation and ECM accumulation through the canonical Smad-dependent and Smad-independent pathways that promote the transcription of collagen I, collagen III, fibronectin, α-smooth muscle actin, TIMP-1 and growth factors such as platelet-derived growth factor, fibroblast growth factor and angiogenic growth factor [126].

Experimental studies showed that miRNAs exhibit important regulatory effects on the TGF-β signaling pathway and, conversely, that TGF-β signaling itself may influence miRNA expression and accelerate miRNA maturation [127]. In vitro studies showed that hyperglycemia induces the upregulation of miR-21 mRNA levels that, in turn, induce Smad7 downregulation and a higher phosphorylation of Smad2 and Smad3 in cardiac fibroblasts, thus promoting myocardial fibrosis [128].

Recent studies demonstrated that miR-155 is also upregulated by high glucose levels and induces the overexpression of Smad-2 [129]. Conversely, miR-15b and miR-141 exert anti-antifibrotic effects by targeting TGFβR1 and TGF-β1, respectively, thus preventing the activation of the fibrotic signaling pathway; miR-15b and miR-141 have been shown to be downregulated by hyperglycemia, with the consequent upregulation of TGFβR1 and TGF-β1.

Besides tight glycemic control, the prevention of cardiac fibrosis and remodeling is a valuable therapeutic strategy in patients with diabetic cardiomyopathy. Pharmacological drugs such as angiotensin-converting enzyme inhibitors, Ang-II receptor blockers and aldosterone antagonists have already proven their efficacy in reducing myocardial fibrosis and improving cardiac function in HF patients. Targeting the TGF- β signaling pathway and mi-RNAs may represent a promising therapeutic intervention in these patients.

## 6. Role of Epicardial Adipose Tissue in DCM

Epicardial adipose tissue (EAT) is a complex endocrine organ with functions that extend beyond just providing warmth and mechanical protection to the heart [130,131]. EAT is a type of visceral fat that is located between the epicardium and the visceral layer of the pericardium. It covers roughly 80% of the surface of the heart [132,133,134]. EAT presents smaller adipocytes that express uncoupling protein 1 (UCP1), typical of brown adipose tissue (BAT) [134,135]. In T2D patients, the uptake of glucose is reduced in BAT, decreasing the expression of peroxisome proliferator-activated receptor-gamma coactivator-1alpha (PGC-1α), which is a key regulator of energy metabolism [136]. Furthermore, EAT contains anti-inflammatory macrophages [137]; it has been demonstrated that EAT in T2D patients shows different gene expression, with a higher expression of genes associated with inflammation and of cytokines. This suggests that diabetes may predispose patients to detrimental cardiovascular effects by altering the inflammatory response and cytokine activity in EAT [138]. Numerous cardiovascular imaging studies have shown that patients with T2D have a significantly increased area/volume of EAT [139,140,141,142,143]. Moreover, studies using invasive EAT biopsies and histological assessments have indicated that in T2D and obesity, there is a shift in the balance of adipocytokines within the EAT. This shift favors pro-inflammatory adipocytokines over anti-inflammatory ones, leading to chronic, low-grade inflammation. This imbalance may contribute to the development of cardiovascular disease [138]. Consistently, imaging and histological studies evidence that EAT acts in a key role in the pathological development of DCM [144].

## 7. Conclusions

**Figure 2:**
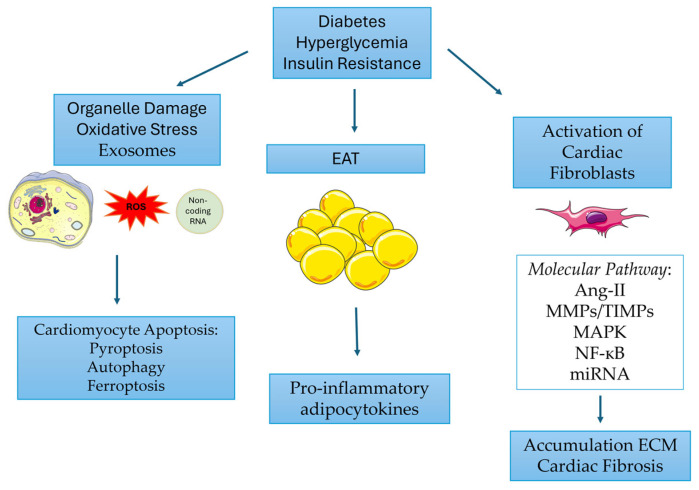
The roles of organelle damage, oxidative stress, exosomes, epicardial adipose tissue (EAT) and activated fibroblasts in DCM. Epicardial adipose tissue (EAT). The figure was generated using Servier Medical Art, provided by Servier, licensed under Creative Commons Attribution 3.0 Unported license. Angiotensin II (Ang-II), matrix metalloproteases (MMPs), tissue inhibitors of metalloproteinases (TIMPs), mitogen-activated protein kinase (MAPK), nuclear factor kappa-light-chain-enhancer of activated B cells (NF-κB), microRNA (miRNA).

DCM represents one of the major diabetes complications. Different mechanisms have been discovered to be the causes of DCM (Figure 2). The main mechanisms have been described in this review, which led to the development of promising therapeutic targets.
